# Evaluation of Fluorescence Intensity and Antitumor Effect Using Real-Time Imaging in Photoimmunotherapy

**DOI:** 10.3390/ph15020223

**Published:** 2022-02-14

**Authors:** Kenji Takashima, Yoshikatsu Koga, Takahiro Anzai, Kayo Migita, Toru Yamaguchi, Akihiro Ishikawa, Shingo Sakashita, Masahiro Yasunaga, Tomonori Yano

**Affiliations:** 1 Department of Gastroenterology and Endoscopy, National Cancer Center Hospital East, Kashiwa 277-8577, Japan; ktakashi@east.ncc.go.jp (K.T.); kmigita@east.ncc.go.jp (K.M.); toryamag@east.ncc.go.jp (T.Y.); akishika@east.ncc.go.jp (A.I.); 2 NEXT Medical Device Innovation Center, National Cancer Center Hospital East, Kashiwa 277-8577, Japan; 3 Department of Strategic Programs, Exploratory Oncology Research & Clinical Trial Center, National Cancer Center, Kashiwa 277-8577, Japan; ykoga@east.ncc.go.jp; 4 Division of Developmental Therapeutics, Exploratory Oncology Research & Clinical Trial Center, National Cancer Center, Kashiwa 277-8577, Japan; taanzai@east.ncc.go.jp (T.A.); mayasuna@east.ncc.go.jp (M.Y.); 5 Shimadzu Corporation, Kyoto 604-8511, Japan; 6 Division of Developmental Pathology, Exploratory Oncology Research & Clinical Trial Center, National Cancer Center, Kashiwa 277-8577, Japan; ssakashi@east.ncc.go.jp

**Keywords:** Photoimmunotherapy, antibody-drug conjugate, real-time imaging, laser irradiation dose

## Abstract

Photoimmunotherapy (PIT) is a promising tumor-selective treatment method that uses light-absorbing dye-conjugated antibodies and light irradiation. It has been reported that IR700 fluorescence changes with light irradiation. The purpose of this study was to investigate the fluorescence intensity and antitumor effect of PIT using real-time fluorescence observation of tumors and predict the required irradiation dose. The near-infrared camera system LIGHTVISION was used to image IR700 during PIT treatment. IR700 showed a sharp decrease in fluorescence intensity in the early stage of treatment and almost reached a plateau at an irradiation dose of 40 J/cm. Cetuximab-PIT for A431 xenografts was performed at multiple doses from 0–100 J/cm. A significant antitumor effect was observed at 40 J/cm compared to no irradiation, and there was no significant difference between 40 J/cm and 100 J/cm. These results suggest that the rate of decay of the tumor fluorescence intensity correlates with the antitumor effect by real-time fluorescence imaging during PIT. In addition, when the fluorescence intensity of the tumor plateaued in real-time fluorescence imaging, it was assumed that the laser dose was necessary for treatment.

## 1. Introduction

Photoimmunotherapy (PIT) is an innovative cancer treatment method that utilizes a monoclonal antibody (mAb) conjugated near-infrared photosensitive substances such as IR700, and light irradiation [1]. During PIT, mAb-IR700 binds specifically to antigens overexpressed in the cancer cell membrane. When exposed to 690 nm light, the physical properties of IR700 change, causing the membrane antigen-antibody complex to denature and aggregate. As a result, the cell membrane is damaged, causing rapid necrosis and cell death [2,3]. PIT has been investigated in basic experiments with various antibodies and cancer cells and has been found to be an innovative therapy that can selectively treat only the cancer cells to which the antibody binds [4,5,6,7,8,9,10,11,12].

In clinical practice, PIT has been most commonly used in head and neck cancer, where cancer is close to the body surface, and light irradiation is easy to perform. Moreover, phase III clinical trials are underway worldwide for patients with head and neck cancer using the EGFR-targeting cetuximab-IR700 (Cet-IR700). In addition, in September 2020, PIT was approved by the Pharmaceuticals and Medical Devices Agency (PMDA) in Japan to treat “unresectable, locally advanced or locally recurrent head and neck cancer” based on the results of a phase I trial ahead of other countries [13].

During PIT, it is important to administer the entire light dose only to the tumor. Therefore, there is a need to develop imaging methods that can accurately detect the extent of the tumor and determine the amount of light irradiation that will produce therapeutic effects. Fluorescence imaging is a method of observing cancer cells in real-time using fluorescent substances, such as IR700, thus enabling the observation of drug distribution in tumors [14,15,16]. LIGHTVISION is a commercially available imaging system for indocyanine green (ICG) and has been used in basic studies of the *super*-enhanced permeability and retention (SUPR) effects of PIT [17]. It was reported that by using LIGHTVISION to detect the fluorescence of IR700 in real time, tumor accumulation of trastuzumab-IR700 antibody could be visualized during treatment, and the location of subcutaneous tumors in mice could be confirmed [18,19,20]. However, few reports have examined the relationship between the therapeutic effects of PIT and fluorescence intensity.

We hypothesized that the therapeutic effect of PIT and fluorescence intensity were correlated because IR700-conjugated antibodies aggregate and induce cell death by light irradiation and simultaneously emit fluorescence. Therefore, in this study, we performed real-time fluorescence imaging of tumors under treatment to investigate the relationship between fluorescence intensity and therapeutic efficacy. In addition, we determined the amount of light irradiation required for effective treatment based on the results.

## 2. Results

### 2.1. Quality Control of Cetuximab-IR700

**Figure 1:**
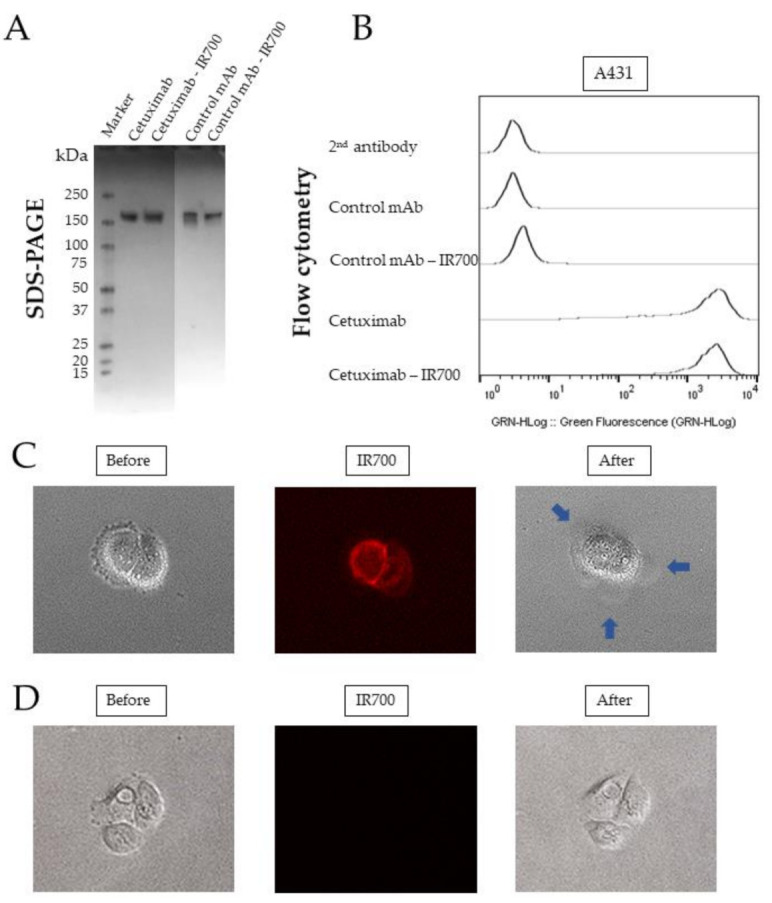
Quality control of Cetuximab-IR700. (**A**) Non-reducing SDS-PAGE analysis of Cet-IR700 and Control mAb-IR700. Marker: molecular mass marker. (**B**) Conjugation of various monoclonal antibodies to A431 cells. Cet-IR700 and cetuximab showed a high binding ability to EGFR protein. (**C**) Morphological changes of cells after photoimmunotherapy (PIT) with Cet-IR700. The EGFR-specific localization of Cet-IR700 in A431 cells was confirmed by fluorescence microscopy. Ruptured A431 cells were observed under the microscope after 20 J/cm^2^ of laser irradiation. Blue arrows indicate cells that have ruptured and formed blisters. (**D**) Morphological changes of cells after PIT with Control mAb-IR700. There was no morphological change of A431 cells before and after PIT, and Control mAb-IR700 could not be observed by fluorescence microscopy.

Cetuximab (chimeric (mouse/human) mAb) and a control mAb (human IgG1 isotype control recombinant clone) were conjugated to IR700 to produce a new antibody drug. The schematic structure of the Cet-IR700 is shown (Figure S1). The produced antibodies, Cet-IR700 and Control mAb-IR700 (negative control), were analyzed by sodium dodecyl sulfate-polyacrylamide gel electrophoresis (SDS-PAGE). Cet-IR700 and non-conjugated cetuximab had the same molecular weight of approximately 150 kDa. The same result was observed in the Control mAb-IR700 (Figure 1A). The conjugation of cetuximab, Cet-IR700, control mAb, and Control mAb-IR700 to A431 cells was examined by flow cytometry. Cet-IR700 and cetuximab showed a high binding ability to the EGFR protein, and the binding ability of EGFR did not change when IR700 was conjugated. In addition, Control mAb-IR700 and control mAb did not bind to the EGFR protein (Figure 1B). Time-lapse fluorescence microscopy imaging was performed to examine the effect of cell membrane damage after PIT and the target-specific localization of Cet-IR700 and Control mAb-IR700. Before treatment, IR700 was mainly localized in the membrane of A431 cells. Immediately after laser exposure, A431 cells formed blisters and ruptured due to cell membrane damage. Most of these morphological changes were observed within 15 min of laser irradiation (Figure 1C). However, Control mAb-IR700 did not localize to the plasma membrane of A431 cells, and the cells did not show any changes after laser irradiation (Figure 1D).

### 2.2. In Vivo Real-Time Fluorescence Imaging during PIT

**Figure 2:**
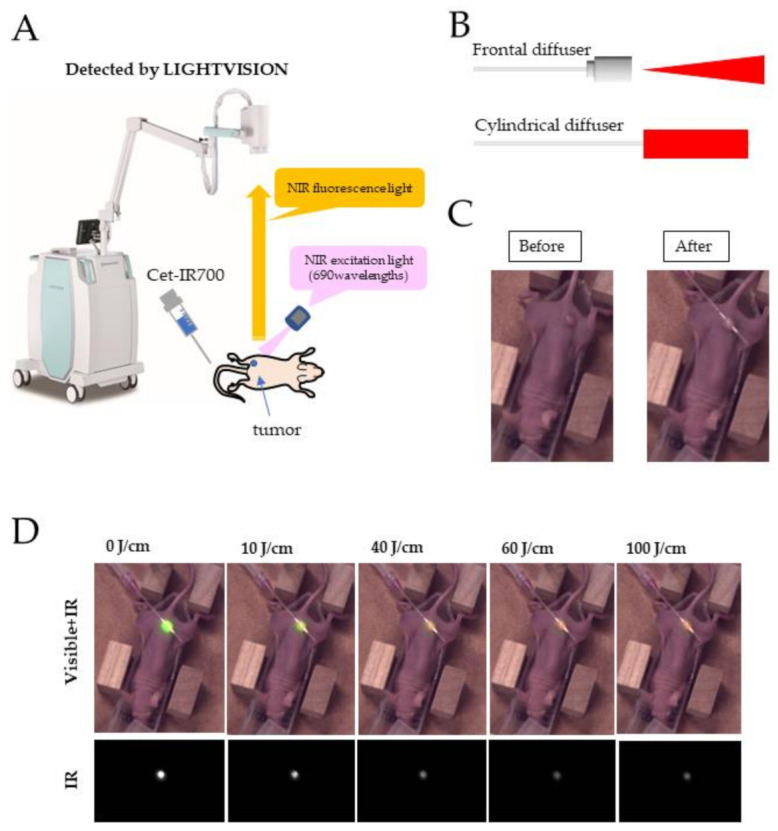
In vivo real-time fluorescence imaging during PIT. (**A**) Experimental setup for fluorescence observation using LIGHTVISION. (**B**) The frontal diffuser provided surface irradiation to tumors and was used for in vitro experiments and tissue evaluation. The unit of irradiation dose was J/cm^2^. The cylindrical diffuser provided intra-tissue irradiation of the tumor and was used for in vivo imaging and antitumor efficacy studies. The unit of irradiation dose was J/cm. (**C**) Cylindrical catheter puncture method for subcutaneous tumors in mice. The subcutaneous tissue proximal to the tumor was punctured. (**D**) Mice with a tumor volume of approximately 100 mm^3^ were treated with 100 μg of Cet-IR700. After 24 h, A431 tumors were irradiated with light of a 690 nm wavelength, and fluorescence images were captured by LIGHTVISION. The fluorescence of Cet-IR700 accumulated in the tumor during laser irradiation was detected.

The LIGHTVISION camera is a system for capturing and imaging the fluorescence of indocyanine green (peak fluorescence: 830 nm). Because the fluorescence spectrum of Cet-IR700 extends past 800 nm, it was assumed that the LIGHTVISION camera could detect IR700 fluorescence. Therefore, we set out to confirm whether the LIGHTVISION camera can detect tumors by fluorescence observation when a 690 nm laser is used to irradiate the tumor. The schema of the fluorescence imaging experiment is shown in Figure 2A. Briefly, a cylindrical catheter was punctured along the bottom or side of the tumor in the mice (not into the tumor), and a diffuser was inserted into the catheter to deliver laser radiation (Figure 2B,C), while a frontal diffuser delivered surface radiation to the tumor (Figure 2B). When the subcutaneous tumors of the mice were irradiated with 690 nm light 24 h after the administration of Cet-IR700 (100 μg/animal), the tumor accumulation of the antibody could be visualized, and the location of the tumor could be confirmed. Next, real-time fluorescence imaging was performed on the tumors bound with Cet-IR700, and the fluorescence images at irradiation levels of 0, 10, 40, 60, and 100 J/cm were compared. The results showed that the fluorescence intensity of the tumor was at a maximum at the beginning of administration and gradually decreased as the laser dose increased (Figure 2D).

### 2.3. Real-Time Fluorescence Imaging Analysis of IR700 

**Figure 3:**
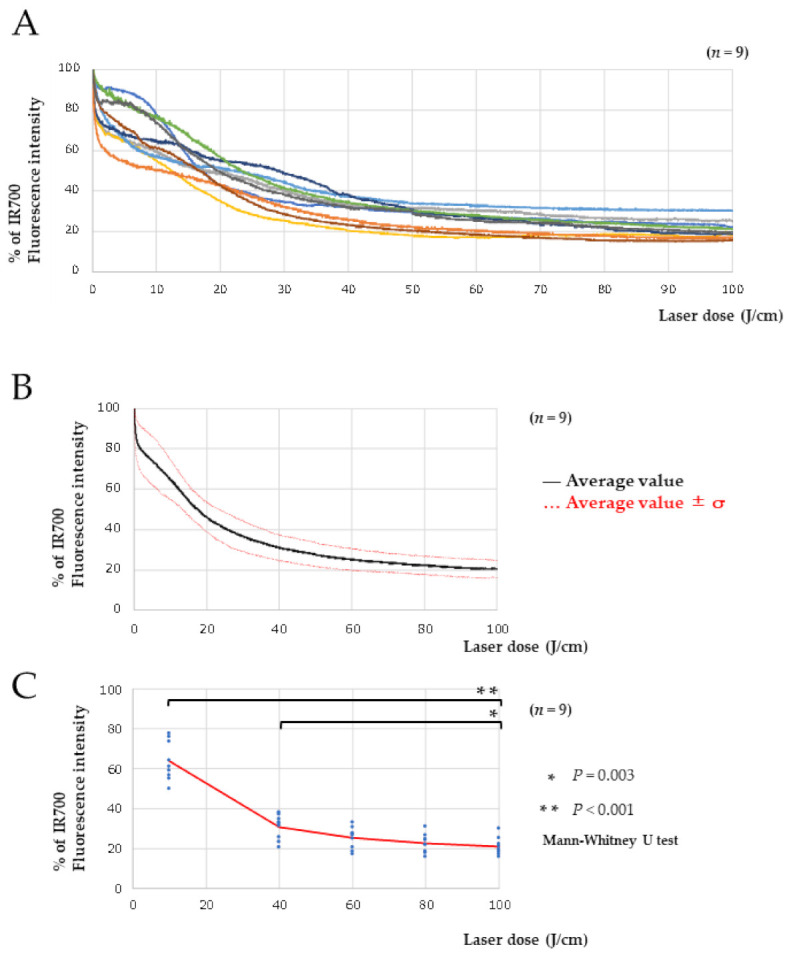
Real-time fluorescence imaging analysis of IR700. (**A**) The time fluorescence intensity curve shows the calculated average fluorescence intensity of Cet-IR700-bound tumors during PIT. (**B**) The mean values of the time fluorescence intensity curves and the mean ± σ (red dots) are shown. (**C**) The rate of decay of fluorescence intensity was compared between the clinical dose of 100 J/cm and the 10, 40, 60, and 80 J/cm groups (*n* = 9, *p* < 0.001, *p* = 0.003, *p* = 0.10, and *p* = 0.51, respectively, analyzed by the Mann–Whitney U test).

Nine mice were treated with PIT, and real-time fluorescence imaging was performed on Cet-IR700-bound tumors. The average fluorescence intensity was calculated by imaging the region of interest of the tumor during treatment, and a graph was created with the *x*-axis corresponding to the laser dosage. Fluorescence intensity analysis of IR700 showed a sharp decrease in the early stages of treatment, and the decrease slowed when the dose reached 40 J/cm (Figure 3A). Averaging the fluorescence intensities of the nine mice, we found that as the radiation dose increased to 60–100 J/cm, the fluorescence intensity decreased to about 20%, almost reaching a plateau (Figure 3B). Comparing the percentage of fluorescence decay at each irradiation dose, the 10 and 40 J/cm groups showed significant differences compared to the 100 J/cm group (*n* = 9, *p* < 0.001 and *p* = 0.003, respectively). The 100, 80, and 60 J/cm groups showed no significant difference, and the decay rate of fluorescence intensity was almost in equilibrium (Figure 3C).

### 2.4. Relationship between Laser Dose and Antitumor Effect

**Figure 4:**
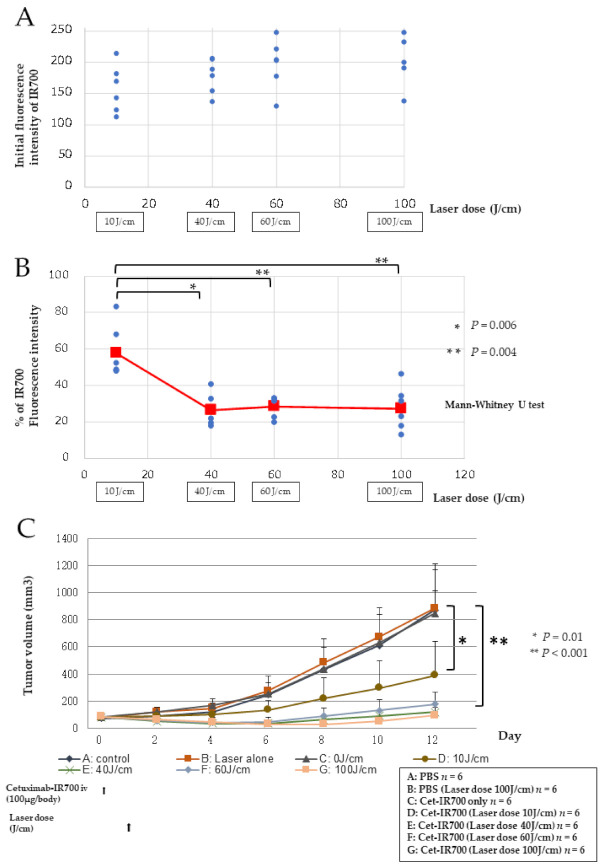
Relationship between laser dose and antitumor effect. (**A**) The fluorescence intensity at the beginning of treatment was examined in each laser dose group (10, 40, 60, 100 J/cm). (**B**) The rate of fluorescence intensity decay at the end of irradiation was examined in each laser dose group (10, 40, 60, 100 J/cm). (**C**) Volumes of A431 tumors were compared among the groups treated with Cet-IR700 and irradiated with various laser doses (10, 40, 60, and 100 J/cm) and the untreated control group. Data are shown as mean values (*n* = 6 per group, 12 days after initial treatment, dots are mean values, bars are SD).

The relationship between the laser dose and the antitumor effect was evaluated. Based on the decay rate of the fluorescence intensity, the laser doses of 10 J/cm, when the fluorescence intensity rapidly declined, 40 J/cm, just before the fluorescence intensity plateaued, and 60 J/cm, when the fluorescence intensity had reached a plateau, were compared with the dose of 100 J/cm. First, we compared the initial fluorescence intensity in each group. The mean value of the fluorescence intensity was 182, and there was no significant difference among the groups, indicating that the antibody accumulated in the tumor (Figure 4A). Next, we examined the rate of decay of the fluorescence intensity at the end of laser irradiation in each group and found a significant difference only between the 10 J/cm and 100 J/cm groups (*p* < 0.004, Mann–Whitney U test, Figure 4B). Using Cet-IR700, PIT was performed at each irradiation dose, and the effect was examined in mice with A431 tumors. The PIT group was irradiated with a laser 24 h after intravenous injection of Cet-IR700. The volume of A431 tumors was significantly reduced in the groups that received Cet-IR700 followed by laser irradiation (10, 40, 60, and 100 J/cm) compared to the no treatment group (*p* < 0.001, *p* < 0.01; one-way analysis of variance, Tukey test). The antitumor effects did not differ significantly among the groups that received 40, 60, and 100 J/cm radiation. In addition, there was a trend of a decrease in tumor volume in the 40, 60, and 100 J/cm dose groups compared to the 10 J/cm dose group (Figure 4C). 

### 2.5. Histological Analysis of PIT

**Figure 5:**
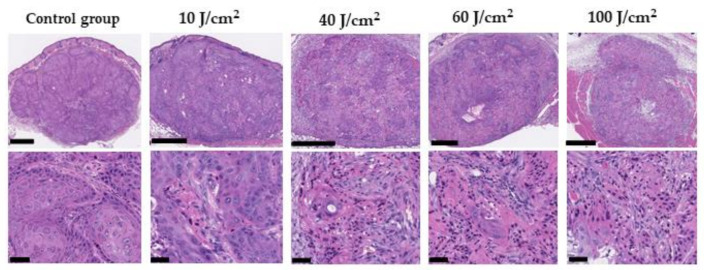
Histological findings 24 h after PIT with various laser doses. Histological specimens of A431 tumors with no treatment and PIT of 10, 40, 60, and 100 J/cm^2^ are shown. All specimens are stained with hematoxylin and eosin. Scattered clusters of damaged tumor cells are seen. (Upper black scale bar = 500 µm, Lower black scale bar = 50 µm).

Histological changes 24 h after PIT with various laser doses were evaluated. Microscopic evaluation of the treated tumors showed scattered clusters of damaged tumor cells after PIT of 10–100 J/cm^2^. The damaged cells had eosinophilic cell chambers and pyknotic nuclei. Although there was no obvious damage in the control group, living but damaged tumor cells were scattered against a background of microhemorrhage and inflammatory cell infiltration consistent with acute granule formation in the laser-irradiated groups. In the groups with a dose of 40 J/cm^2^ or higher, there was no obvious difference in the remaining tumor cells according to the laser irradiance. However, the number of damaged tumor cells in the 10 J/cm^2^ group was lower than that of the other radiation doses (Figure 5). 

## 3. Discussion

PIT is currently in need of an imaging method capable of confirming the location of the tumor and determining the effect of treatment. Upon light irradiation, IR700-conjugated antibodies form aggregates and cause membrane damage in cancer cells. As a result, cell death is induced. In addition, after this photochemical reaction, IR700 does not emit fluorescence due to structural changes, and the fluorescence disappears [2,3]. In our study, PIT treatment of A431 also resulted in membrane damage and loss of IR700 fluorescence after treatment (Figure 1C). And in vivo experiments showed that even if the amount of antibody accumulation in the tumor is high at the beginning of treatment, it does not necessarily mean that the treatment is highly effective (Figure S2). Not only the amount of antibody accumulation, but also the amount of light and the method of irradiation are important to enhance the therapeutic effect. Based on the above, we hypothesized that real-time fluorescence imaging of tumors under PIT treatment and measurement of fluorescence intensity could provide information on the treatment effectiveness of PIT.

In this study, we performed real-time fluorescence imaging of IR700 using commercially available LIGHTVISION, commonly used for ICG. Because the fluorescence peak of IR700 (702 nm) is more than 100 nm from the fluorescence peak of ICG (805–845 nm) [21], the imaging equipment for ICG was not sensitive enough for IR700, proving to be a challenge for fluorescence imaging of IR700. Nevertheless, the location of the tumor was clearly identified in this study (Figure 2D). Although the wavelengths were far from the fluorescence peak of IR700, the absence of collision with the excitation light (690 nm) and the low influence of autofluorescence were considered to have led to good fluorescence imaging of IR700. We also succeeded in performing real-time fluorescence imaging of the tumor during PIT therapy and found that the fluorescence gradually disappeared (Figure 2D). Analysis of IR700 during treatment showed that the fluorescence intensity decreased sharply initially, and from approximately 50 J/cm, the decay of fluorescence intensity reached near-equilibrium (Figure 3A). Therefore, it was expected that under conditions of rapidly changing fluorescence intensity, active cancer cell membrane damage would occur, and under equilibrium conditions of fluorescence intensity, cancer cell membrane damage would decrease. From the results shown in Figure 3A, the laser dose was determined by the rate of fluorescence decay. Then, Cet-IR700 was selectively accumulated in A431 tumors, and the antitumor effect was examined at various irradiation doses. The low dose of 10 J/cm showed a significant antitumor effect compared to no treatment, but the therapeutic effect was insignificant compared to the dose of 40–100 J/cm, where the fluorescence intensity reached equilibrium. Moreover, there was no significant difference in the antitumor effect at doses of 40–100 J/cm. These results further confirm the relationship between the decay rate of fluorescence intensity and the antitumor effect (Figure 4B,C). With respect to the irradiation dose, it was observed that dose-dependent cell death occurred when the fluorescence intensity decreased; however, when the fluorescence intensity decay reached equilibrium, the antitumor effect did not change regardless of the irradiation dose. Therefore, in addition to avoiding side effects induced by high irradiance, it has been reported that irradiance of 50 J/cm^2^ is suitable for inducing the SUPR effect [22,23,24]. It is a technical application to monitor the laser dose during PIT. 

This study has several limitations. First, we only examined the feasibility of this method for a mouse cell line-derived xenograft model. The use of the patient-Derived Xenograft Model would provide a more similar experiment to the human condition. Second, the average tumor size we studied was approximately 100 mm^3^, which does not reflect the repertoire of different tumor sizes. Fluorescence imaging may result in variable fluorescence intensity measurements depending on the tumor type and size. Because the intensity of light decreases through tissue, if the tumor is thick or large, it may not be possible to measure the fluorescence of IR700 binding antibodies that accumulate in the tumor. However, compared to previously reported fluorescence imaging studies of PIT, our study was performed under conditions of reduced light attenuation by irradiating the tumor with intra-tissue irradiation rather than surface irradiation [17,18,19,20]. Third, we used LIGHTVISION, a fluorescence imaging device that detects ICG, and obtained good results. However, similar ICG imaging devices are available [25,26], but we did not study the differences between these devices.

## 4. Materials and Methods

### 4.1. Cells and Cell Culture 

The A431 cell line used in this experiment was purchased from the American Type Culture Collection (Manassas, VA, USA). These cell lines were cultured in Dulbecco’s Modified Eagle Medium (DMEM) (FUJIFILM Wako Pure Chemical Corporation, Osaka, Japan) supplemented with 10% fetal bovine serum (FBS, Thermo Fisher Scientific, Waltham, MA, USA) and 1% penicillin-streptomycin-amphotericin B suspension (FUJIFILM Wako Pure Chemical Corporation, Osaka, Japan) at 37 °C in a 5% CO_2_ atmosphere.

### 4.2. Synthesis of IR700-Conjugated Antibodies

The antibody used was cetuximab (Merck Biopharma, Tokyo, Japan), a human/mouse chimeric monoclonal antibody of the IgG1 subclass that targets EGFR. Ultra-LEAF™ Purified Human IgG1 Isotype Control Recombinant (BioLegend, San Diego, CA, USA) was used as a control antibody. The photosensitive material used was IRDye 700DX NHS Ester (IR700; C74H96N12Na4O27S6Si3, molecular weight: 1954.22), purchased from LI-COR Bioscience (Lincoln, NE, USA). All reagent-grade chemicals were used in this study. The IRDye^®^ 700DX Protein Labeling Kit (LI-COR Bioscience, Lincoln, NE, USA) was used to bind IR700 to the antibody according to a previously established method [27,28]. Briefly, 1 mg (6.6 nmol) of cetuximab was incubated with 53.2 µg (27.2 nmol) of IR700. The mixture was then purified on a Zeba^TM^ Desalting Spin Column, 7K MWCO (Thermo Fisher Scientific), to produce Cet-IR700. The control antibody was prepared in the same way to produce Con-IR700. The number of fluorophore molecules bound to each antibody molecule was determined using the following method. A spectrophotometer (NanoDrop One; Thermo Fisher Scientific) was used to measure the concentration of protein and IR700 by measuring the absorption at 280 nm and 689 nm. On average, two IR700 molecules were bound to each antibody. Non-reducing SDS-PAGE was used to analyze the resulting conjugated antibodies.

### 4.3. Flow Cytometry

Flow cytometry was conducted as previously described [29,30]. Cultured cells were stained using primary antibodies (cetuximab, Cet-IR700, control antibody, Control mAb-IR700) and Alexa Fluor 488-conjugated anti-rat as secondary antibodies. Stained cells were analyzed using Guava easyCyte 10HT (Merck Millipore, Billerica, MA, USA). The data were analyzed using FlowJo software (Tree Star Inc., Ashland, OR, USA).

### 4.4. In Vitro Fluorescence Microscopic Images and PIT 

Fluorescence microscopy was performed using a BZ-X700 fluorescence microscope (Keyence, Osaka, Japan). The molecular target-specific localization of Cet-IR700 and Control mAb-IR700 was confirmed in A431 cells. Cells were seeded on 96-well optical-bottom plates with a polymer base (Thermo Fisher Scientific) and incubated at 37 °C for 24–48 h. Cet-IR700 (10 μg/mL) or Control mAb-IR700 (10 μg/mL) was added to the culture medium and incubated for 3 h at 37 °C. The cells were then washed with phosphate-buffered saline (PBS) and observed under a fluorescence microscope. Next, they were laser irradiated with a 690 nm continuous wave laser (MLL-III-690; CNI Optoelectronics Technology, Changchun, China). A power density of 30 mW/cm^2^ was measured using an optical power meter (PM100, Thorlabs, Newton, NJ, USA). The laser dose was 20 J/cm^2^. The cells before and after irradiation were observed under a microscope.

### 4.5. Animal Model

Six-week-old female BALB/c nu/nu mice (Charles River Japan, Yokohama, Japan) were purchased. Mice were anesthetized with isoflurane and inoculated with 3.5 × 10^6^ A431 cells suspended in 100 μL of PBS on the left dorsal side. Tumor volume was calculated using the following equation: TV = (L × W^2^)/2, where L and W are the length and width of the tumor under the skin, respectively [31]. Tumor volume and weight were measured every two days, and mice that reached 1000 mm^3^ or lost more than 20% of their body weight were euthanized due to humane treatment. Animal experiments were approved by the Animal Experiment Committee of the National Cancer Center Japan. All animal experiments were performed in accordance with the Guidelines for the Care and Use of Laboratory Animals established by the committee. These guidelines meet the ethical standards set forth by law and comply with the Japanese Guidelines for the Use of Laboratory Animals.

### 4.6. In Vivo Real-Time Fluorescence Imaging

To perform fluorescence imaging during PIT, LIGHTVISION (Shimadzu Corporation, Kyoto, Japan) was used at a collection wavelength of 820 nm or higher, and the excitation light was a 690 nm laser (150 mW/cm) from a laser irradiation device (MLL-III-690) used in PIT therapy. A cylindrical diffuser, which emits light from the laser device to the tumor, was placed alongside the tumor (no diffuser was placed inside the tumor), and intra-tissue irradiation was performed. The LIGHTVISION camera head was placed approximately 50 cm from the observation target. Mice with A431 tumors of approximately 100 mm^3^ were injected with 100 μg of Cet-IR700, and 24 h later, PIT treatment was performed to obtain fluorescence images and tumor fluorescence intensity during treatment. The exposure time, camera sensitivity, binning, and other conditions were observed under the same settings, and fluorescence imaging was performed continuously. The obtained fluorescence images were analyzed by Fiji software [32]. After setting the region of interest for each tumor region, the pixel values were measured, and a graph was created with the x-axis corresponding to the light intensity. All pixel values were normalized to a maximum intensity of 100%.

### 4.7. In Vivo PIT with Cet-IR700

In vivo experiments were performed using mice implanted with A431 cells. Mice with A431 tumor xenografts and a tumor volume of approximately 100 mm^3^ were selected and randomly divided into seven groups (*n* = 6 mice per group). The seven groups were as follows: (a) No treatment (intravenous injection of PBS without laser irradiation). (b) Laser irradiation (100 J/cm 24 h after intravenous injection of PBS. (c) Intravenous injection of 100 µg of Cet-IR700 without laser irradiation. (d) Laser irradiation (10 J/cm) 24 h after intravenous injection of 100 µg Cet-IR700. (e) Laser irradiation (40 J/cm) 24 h after intravenous injection of 100 µg Cet-IR700. (f) Laser irradiation (60 J/cm) 24 h after intravenous injection of 100 µg Cet-IR700. (g) Laser irradiation (100 J/cm) 24 h after intravenous injection of 100 µg Cet-IR700. Laser irradiation was performed under isoflurane anesthesia, and a 690 nm continuous wave laser was used at a power density of 150 mW/cm. In addition, a cylindrical diffuser was placed near the tumor (the diffuser was not inserted inside the tumor), and intra-tissue irradiation was performed. After PIT treatment, the tumor volume was measured once every two days until the tumor volume reached 1000 mm^3^.

### 4.8. Histological Analysis

A series of histological changes 24 h after PIT with various laser doses were evaluated. Mice with A431 tumors were euthanized after 24 h of irradiation at 40, 60, and 100 J/cm^2^. Laser irradiation was performed using a frontal diffuser to avoid any physical effects of the diffuser on the tumors, and a 690 nm (150 mW/cm^2^) laser was used. The tumors were then excised, fixed in 10% formalin, and embedded in paraffin. Serial sections (4 μm) were fixed on glass slides and stained with hematoxylin and eosin. Pathological images were analyzed using Nano Zoomer 2.0HT (Hamamatsu Photonics, Hamamatsu, Japan) and the image viewing software NDP.view2 (Hamamatsu Photonics). 

### 4.9. Statistical Analysis

Statistical analyses were performed using EZR, a modified version of R commander designed to add statistical functions frequently used in biostatistics [33]. For multiple comparisons with the control group, one-way analysis of variance. The Mann–Whitney U test was used to compare the decay rate of fluorescence intensity with the amount of laser irradiation. Statistical significance was set at *p* < 0.05.

## 5. Conclusions

In conclusion, we have successfully visualized target tumors during PIT using Cet-IR700 with LIGHTVISION. Analysis of the fluorescence intensity of tumors during PIT showed a relationship between the decay rate of the fluorescence intensity and the antitumor effect. As for the laser dose, it was suggested that dose-dependent cell death occurs when the fluorescence intensity decays; however, when the fluorescence intensity decay reaches equilibrium, the therapeutic effect may remain the same regardless of the dose.

## Data Availability

The data that support the findings of this study are available from the corresponding author, [Tomonori Yano], upon reasonable request.

## Supplementary Materials

The following supporting information can be downloaded at: https://www.mdpi.com/article/10.3390/ph15020223/s1, Figure S1: Schematic structures of Cet-IR700, Figure S2: Relationship between Initial fluorescence intensity of IR700 and antitumor effect.
