# Supplementary material for: Evaluation of Fluorescence Intensity and Antitumor Effect Using Real-Time Imaging in Photoimmunotherapy
Source: Pharmaceuticals (Basel). 2022 Feb 14;15(2):223. doi: 10.3390/ph15020223 (PMC8880675; doi:10.3390/ph15020223)
Supplement: Supplementary file 1 [file pharmaceuticals-15-00223-s001.zip › pharmaceuticals-1577000-supplementary.pdf]

Supplementary Figures

Supplementary Figure S1.

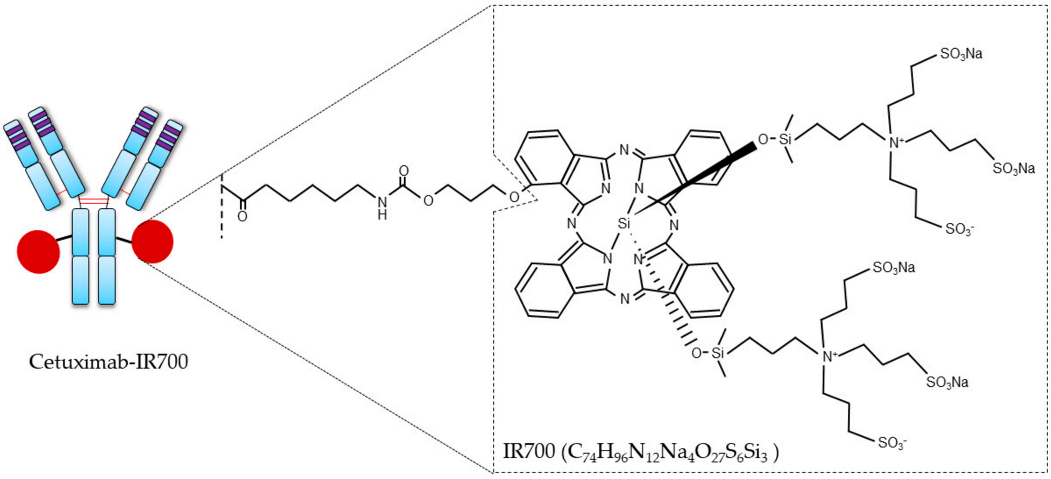

Supplementary Figure S1. Schematic structures of Cet-IR700.

Cetuximab was conjugated to IR700 to produce a new antibody drug. An average of two IR700 molecules are covalently conjugated to cetuximab.

Supplementary Figure S2.

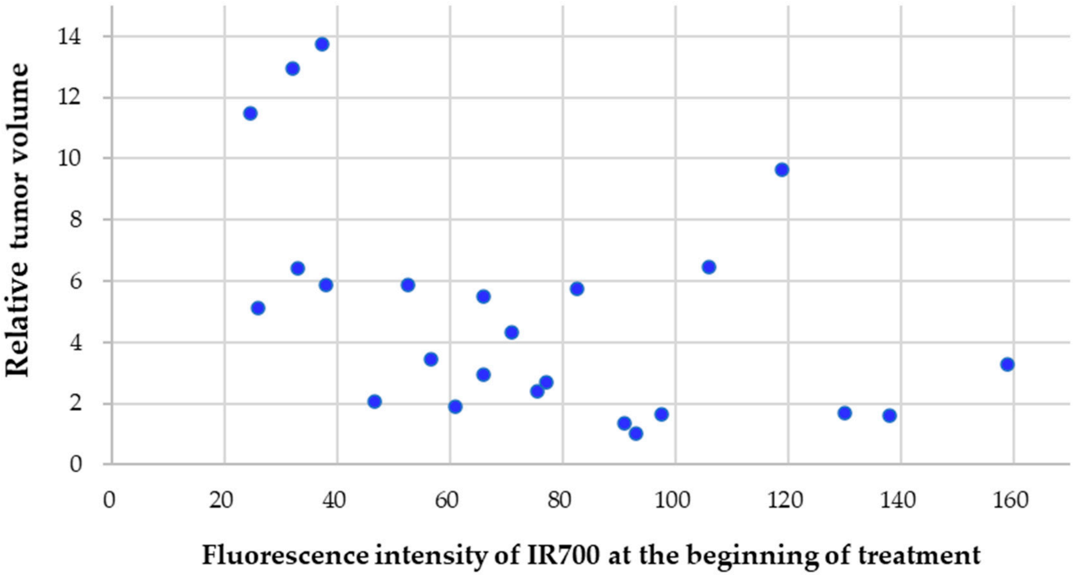

Supplementary Figure S2. Relationship between Initial fluorescence intensity of IR700 and antitumor effect.

The fluorescence intensity at the beginning of treatment was examined in each Cet-IR700 group (Cet-IR700 dose: 30, 50, 70, and 100 μg/animal). In addition, laser irradiation (100 J/cm) was performed 24 hours after the administration of Cet-IR700, and the relationship between the fluorescence intensity at the beginning of treatment and the volume of A431 tumor after 14 days was evaluated (n=6 per group). Some mice showed tumor growth even when the initial fluorescence intensity was high (119,106). On the other hand, some mice showed suppressed tumor growth even when the initial fluorescence intensity was relatively low (61, 47). These results suggest that a high accumulation of antibodies at the beginning of treatment does not necessarily mean a high therapeutic effect. In order to enhance the therapeutic effect, not only the antibody accumulation but also the amount and delivery of light are important.
